# An Advanced Lung Carcinoma Prediction and Risk Screening Model Using Transfer Learning

**DOI:** 10.3390/diagnostics14131378

**Published:** 2024-06-28

**Authors:** Isha Bhatia, Aarti, Syed Immamul Ansarullah, Farhan Amin, Amerah Alabrah

**Affiliations:** 1 Department of Computer Science and Engineering, Lovely Professional University, Phagwara 144001, India; ishabhatia91@gmail.com (I.B.); aarti.1208@gmail.com (A.); 2 Department of IMBA (Integrated Master of Business Administration), North Campus Delina, The University of Kashmir, Srinagar 190001, India; syedansr@gmail.com; 3 School of Computer Science and Engineering, Yeungnam University, Gyeongsan 38541, Republic of Korea; 4 Department of Information Systems, College of Computer and Information Science, King Saud University, Riyadh 11543, Saudi Arabia

**Keywords:** lung carcinoma, CT image, deep learning (DL), machine learning (ML)

## Abstract

Lung cancer, also known as lung carcinoma, has a high death rate, but an early diagnosis can substantially reduce this risk. In the current era, prediction models face challenges such as low accuracy, excessive noise, and low contrast. To resolve these problems, an advanced lung carcinoma prediction and risk screening model using transfer learning is proposed. Our proposed model initially preprocesses lung computed tomography images for noise removal, contrast stretching, convex hull lung region extraction, and edge enhancement. The next phase segments the preprocessed images using the modified Bates distribution coati optimization (B-RGS) algorithm to extract key features. The PResNet classifier then categorizes the cancer as normal or abnormal. For abnormal cases, further risk screening determines whether the risk is low or high. Experimental results depict that our proposed model performs at levels similar to other state-of-the-art models, achieving enhanced accuracy, precision, and recall rates of 98.21%, 98.71%, and 97.46%, respectively. These results validate the efficiency and effectiveness of our suggested methodology in early lung carcinoma prediction and risk assessment.

## 1. Introduction

Lung carcinoma is one of the most dangerous types [1] of cancer compared to the others, including prostate, breast, and colon cancer [2,3,4,5]. Lung carcinoma is categorized into two types: non-smallcell and smallcell. Both types are caused by smoking, but people who have never smoked in their lives are also affected by the disease. There are other causes, including air pollution, infected water, and hazardous gasses. To cure a patient ofthe disease, accurate identification of non-small cell lung cancer at stage I or stage II is essential. Detecting lung cancer is not a difficult task, and identifying it early is vital. Even with advancements in technology, early detection of lung cancer is still challenging [6]. Many patients are diagnosed in later phases when treatment options are limited. Predicting lung cancer at an early stage to identify high-risk individuals is becoming a pressing need. Researchers are looking at automating the analysis of medical images by using advanced computational methods such as deep learning (DL) and machine learning(ML) to improve accuracy [7,8]. These techniques can help speed up the detection process and enhance accuracy. Transfer learning is a particularly helpful technique because it enables computers to better recognize and analyze medical images using information from massive datasets [9,10].

In the literature, researchers have reported the use of this technology. For example, ref. [11] presented information on lung cancer segmentation from computed tomography (CT) images [12]. The proposed method involves integrating data from neighboring CT slices combined with discrete wavelet transform [13,14,15]. They suggested deep learning supervision to provide a more comprehensive textural analysis [16,17]. Similarly, Hussain et al. [18] developed a lung cancer prediction system that extracts gray-level co-occurrence matrix (GLCM) features from improved photos by applying and perfecting powerful ML classification algorithms. The key findings of the authors showed that the proposed model might be highly useful in enhancing lung cancer prognoses by expert radiologists, lowering the death rate, even though the proposed model’s effectiveness was hampered. There is no therapeutically equivalent technique in the literature, and the current literature still has the following drawbacks.

1. Lung carcinoma detection mainly depends on invasion into the bronchioles and ribs along with abnormal growth. Therefore, prediction at an early stage is a difficult task due the need to identify these drastic changes.
2. The quality of CT images affects the accuracy of the classification process.
3. Preprocessing lung CT images shows incoherent negative findings due to the low edge enhancement and sequential processing.
4. By commonly segmenting the region of interest (ROI) from lung images, extracted features are given a high score based on a nearby class, and the method is not effective for detection.

In this research, we proposed an advanced prediction model for predicting lung cancer at an early stage. Our strategy improves early lung cancer detection efficiency and accuracy, which is critical for better patient outcomes.

The working mechanism of our proposed model is as follows.

1. **Preprocessing:** To enhance image quality and make additional analysis easier, lung computed tomography images are first preprocessed. This involves multiple steps.
  - *Noise Reduction*: We remove noise from CT images by applying a sophisticated improved anisotropic diffusion filter (I-ADF) approach.
  - *Contrast stretching*: Enhancing the contrast of the images makes important features more distinguishable.
  - *Convex Hull Lung Region Extraction*: In order to concentrate the analysis on the pertinent area, we isolate the lung region.
  - *Edge Enhancement*: Using the unsharp masking filter (UMF) improves the ability to define the image edges.
2. **Segmentation:** The Bates distribution coati optimization (B-RGS) algorithm segments the preprocessed pictures for feature extraction. Important elements that are essential for precise categorization are removed from the segmented images.
3. **Classification:** The images are categorized as either normal or abnormal using the PResNet classifier. If the output is abnormal, thorough risk screening is carried out to ascertain whether the cancer risk is low or high. This multi-step process guarantees a complete evaluation with precise predictions.

The key contributions of the proposed model are listed below.

1. We propose an advanced lung carcinoma prediction and risk screening model using transfer learning.
2. The I-ADFtechnique eliminates noise from the CT image.
3. Our B-RGS algorithm is powered by segmentation and helps partition the lungs by using knuckle points.
4. Our algorithm improves edge enhancement by using the UMF.
5. The model’s accuracy is achieved through feature selection.

The format of this paper is as follows. A summary of previous approaches is given in Section 2. The proposed model is discussed in Section 3, and the outcomes of the suggested methodology are discussed in Section 4. The conclusions are in Section 5.

## 2. Related Work

With an astounding five million fatalities every year, lung cancer ranks among the leading causes of death worldwide for both men and women. A CT scan can yield important data for lung disease diagnosis. This work’s primary goal is to identify cancerous lung nodules from lung images and to categorize lung cancer according to severity. Using cutting-edge deep learning techniques, this work locates malignant lung nodules [19].

- **Background and Rationale**

Early cancer detection is possible by radiologists, so oncologists are seeing a rise in early lung cancer detection thanks to CT images. For patients with lung cancer, CT imaging can be used to assess various parameters [20,21] like the size, location, and type of lung lesion as well as to offer details on the morphological manifestation [22,23,24]. However, there is a need to perform much more in the medical pitch of lung malignancy [2,25,26,27,28], particularly in terms of early identification and screening [29,30] so that risk factors can be reduced accordingly [31,32,33] and lives can be saved. To reduce mistakes and ensure the accurate segmentation of tumors and organs, the tumors and healthy tissue must not be over or under-irradiated [34,35,36]. By breaking an image into smaller groups using the segmentation approach [37,38,39], its complexity is diminished. Segmentation was previously carried out manually, which can produce unreliable and unstable results. However, when compared to outcomes obtained through human segmentation, automatic techniques such as region-expanding or multi-seed methods boost repeatability and deliver higher-quality radiomic characteristics [40].

- **Machine Learning**

The goal of ML, an artificial intelligence subfield, is to develop algorithms that can solve issues without the need for explicit programming [2,41]. Specialized features for quantitative descriptions of images, the identification of biomarkers for response evaluation, and the prediction of clinical outcomes have all been developed using traditional machine learning techniques [42]. Typical machine learning to identify lung cancer includes the following:

◦ Decision trees (DTs): These are helpful in building models for decision-making that are easy to understand.
◦ Support vector machines (SVMs): These are useful for classification applications [11,19].
◦ Bayesian networks: These are an example of a probabilistic graphical model.

- **Specific ML Models and Techniques**

A risk prediction model [43] was used to identify high-risk people by using CT lung malignancy screening in China. This model demonstrated sufficient biased exactness and is composed of predictors that are widely accessible or readily available in a general large screening situation. However, the model’s dataset was self-reported and prone to measurement error.

The authors of [43] proposed machine learning techniques to create effective models to identify those at a high risk of developing lung carcinoma and, consequently, to implement interventions to prevent it. The outcomes demonstrated improved lung cancer detection. However, because of privacy concerns, accessing critical medical data from the model is challenging [44].

The classification of lung malignancies was improved by recovering characteristics from CT scan images of the damaged regions with noise removed by using an enhanced deep neural network (NN) [45,46]. But the accuracy of the model decreased [47,48,49].

- **Deep Learning Techniques**

With better screening and early diagnosis, DL techniques have been applied more and more to handle huge and complicated datasets, leading to a considerable reduction in lung cancer mortality [6,15,20,50]. These techniques work well for situations involving a lot of calculation and repeated work, which is why medical image analysis uses them.

◦ Convolutional Neural Networks (CNNs)
  a. Classification of Early-Stage Lung Cancer
  The potential for categorizing several forms of lung cancer, including squamous cell carcinoma and adenocarcinoma, has been demonstrated using a deep learning model that was trained on a dataset of 311 individuals with early-stage lung cancer. The dataset’s small sample size, however, limited the model’s performance [10,38,51].
  b. Improved Dense Clustering
  This method partitions the afflicted region into parts according to pixel similarity by using a neural network trained with deep learning. Although it is a reliable predictor of lung cancer, it does not prioritize risk assessment [52].
  c. Ensemble CNN Models
  Classification can be enhanced by combining several CNN models. The computing expenses associated with this ensemble technique are substantial [44,53,54,55].

- **Regression Neural Networks**

For juxta vascular and juxta pleural illnesses, regression neural networks place a strong emphasis on precise lung parenchyma segmentation and distinct boundary detection. This method works well for identifying nearby lesions that are comparable in intensity, which lessens the difficulty of identifying involuntary lesions [55].

- **Artificial Intelligence**

Artificial intelligence (AI) now allows for the analysis of large volumes of diagnostic imaging with increased precision to boost the effectiveness of the healthcare system [3]. Technological developments and computer improvements, new AI algorithms, and neural artificial networks for diagnosis can be trained on hardware. As for proficiency with a variety of scan types, comprehensive understanding already exists, and machines are able to train computers to evaluate and compare models. A massive amount of data from cancer scans provide exceptional diagnostic abilities. Software programs have been assessed and contrasted with the diagnostic instruments used by traditional cancer doctors. Their accuracy has significantly increased, and they are thought to be very helpful in early cancer diagnosis and long-term cancer predictions. Scientists are now using AI systems that have the potential to diagnose breast cancer far earlier and with prognoses that may exceed those of physicians. Informatics also created an AI algorithm and advanced learning systems that might predict who uses low-dose CT analysis to develop lung cancer scans. CNNs have recently been used to diagnose stomach cancer based on invasion depth using gastric endoscopy.

- **Weaknesses and Gaps**
  ◦ Limited Accuracy: Despite innovations, achieving a high accuracy remains difficult for present methods, especially for early-stage detection where small irregularities can go undetected.
  ◦ Subjectivity and Interpretation: Radiologists’ subjective bias and time-consuming manual analysis of medical images might result in inconsistent diagnoses.
  ◦ Data Availability: The lack of labeled medical datasets for DL model training often restricts the creation of reliable and useful algorithms.
  ◦ Complexity and Computational Cost: Some revolutionary methods may be difficult for real-time medical use because of their high computational resource requirements.
- **The Need for our Proposed Work**
  - ***Addressing Limitations:***
    ◦ Enhanced Accuracy: By using transfer learning and advanced preprocessing techniques, the proposed method overcomes the inadequacies of existing methods and increases the accuracy of lung cancer detection.
    ◦ Objective and Automated Analysis: The suggested model reduces the need for arbitrary human interpretation by automating analysis, which produces diagnoses that are more consistent and trustworthy.
    ◦ Optimization for Healthcare: The model’s computational efficiency is improved to make it appropriate for applications that need real-time data.
  - ***Innovations and Contributions***
    ◦ Segmentation Algorithm: Using the B-RGS algorithm to segment lung regions is a revolutionary approach that advances the field and increases feature extraction accuracy.
    ◦ Comprehensive Evaluation: Using standard datasets, several studies are carried out to compare the performance of the suggested method against those of the most recent methods.
    ◦ Demonstrated Quality: The experimental findings show that the suggested model works better than state-of-the-art techniques in terms of accuracy, precision, and recall, underscoring its potential applications in healthcare.

## 3. Risk Screening for Lung Carcinoma

**Figure 1:**
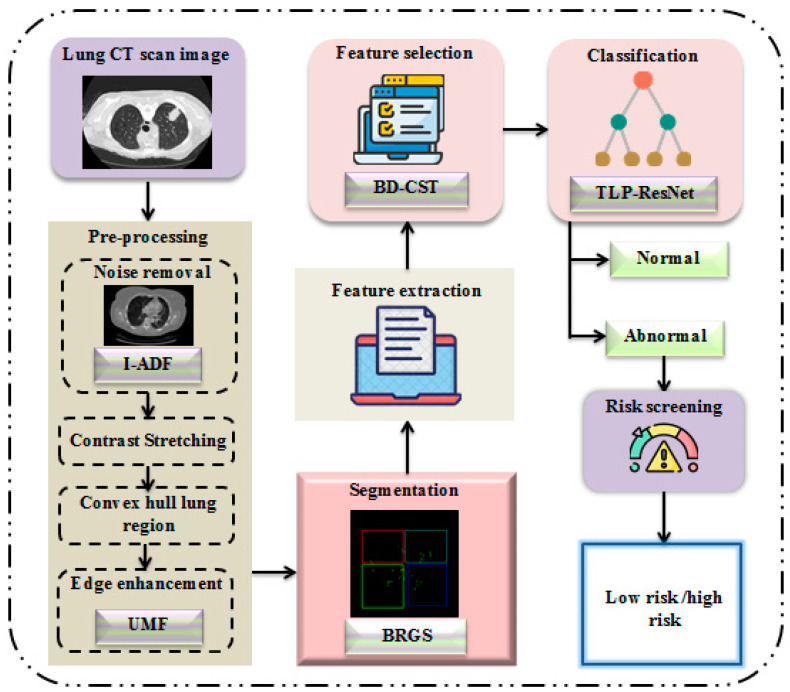
Proposed approach.

Our proposed methodology goes through the following steps: preprocessing, segmentation, feature extraction, feature selection, classification, and risk screening. A block diagram is shown in Figure 1, with the explanation of each setup given below. 

### 3.1. Preprocessing

Initially, the CT image, (I), from a publicly available dataset [44] is preprocessed to obtain a better value from it. During preprocessing, the various steps are noise removal, contrast stretching, convex hull lung region extraction, and edge enhancement. 

***Noise Removal:*** We apply an improved anisotropic diffusion filter to reduce image noise while preserving important edges. If the input image (I) contains noise (e.g., Gaussian or Poisson), the noise should be removed. The ADF is used to eliminate distortion and noise from an image without blurring any of the edges. However, the ADF has a constant diffusion magnitude that affects the signal-to-noise ratio (SNR). To overcome this problem, we propose intra-class variance by replacing local variance. Hence, the proposed method is improved and named the I-ADF.

Let the diffusion matrix, $\left(D_{mt}\right)$, that shares the eigenvectors with eigenvalues related to the level of noise be defined as follows:

(1) $$\alpha_{1}=1-C$$

(2) $$\alpha_{2}=1-C+\frac{3}{2}\left(1-C_{planar}\right)$$

(3) $$\alpha_{3}=1-C+\frac{3}{2}\left(1-C_{planar}\right)+3\left(1-C_{linear}\right)$$

where $\alpha_{1}$, $\alpha_{2}$, and $\alpha_{3}$ denote the eigenvalues, C represents the gain coefficient, and $C_{planar}$ and $C_{linear}$ represent the gain in the local planar neighborhood and thelocal linear neighborhood, respectively:

(4) C=C(〈I〉,Var(I))

(5) $$C_{planar}=C\left({\langle I\rangle }_{e2,e3},Var{\left(I\right)}_{e2,e3}\right)$$

(6) $$C_{linear}=C\left({\langle I\rangle }_{e2,e3},Var{\left(I\right)}_{e2,e3}\right)$$

where $e_{k},k\in \left[1,b\right]$ refers to the eigenvectors, 〈I〉 represents the local mean assessment of image (I), and Var(I) represents intra-class variance, which is calculated as follows:

(7) $$Var\left(I\right)={\sum \left(I-\bar{I}\right)}^{2}$$

where $\bar{I}$ denotes the average of I, with ${\langle I\rangle }_{e2,e3}$ and ${\langle I\rangle }_{e3}$ being local mean values of I, which are computed at voxel position z as follows:

(8) $${\langle I\rangle }_{e2,e3}=\frac{1}{25}\sum_{b,c}I\left(z+be_{2}+ce_{3}\right)$$

(9) $${\langle I\rangle }_{e2,e3}=\frac{1}{7}\sum_{b}I\left(z+be_{3}\right)$$

Therefore, the diffusion matrix is expressed as

(10) $$D_{mt}=\left[\begin{array}{ccc} \alpha_{1} & 0 & 0\\ 0 & \alpha_{2} & 0\\ 0 & 0 & \alpha_{3} \end{array}\right]$$

The corresponding diffusion equation is written as the sum of three diffusion terms:

(11) $$\frac{\partial I\left(z,t\right)}{\partial t}=div\left(D_{mt}\nabla I\right)$$

(12) $$=6\left[\frac{1}{6}div\left(\left(1-C\right)\nabla I\right)+\frac{1}{4}div\left(\left(1-C_{planar}\right)\nabla_{planar}I\right)+\frac{1}{2}div\left(\left(1-C_{linear}\right)\nabla_{linear}I\right)\right]$$

where $\nabla_{planar}I$ depicts the projection of a gradient in the plane formed by $\left(e_{2},e_{3}\right)$, $\nabla_{linear}I$ is the projection of the gradient in the direction of $e_{3}$, and ∇I represents the projection of the gradient of (I). Finally, the image with noise removed is obtained and denoted $N_{rem}$. After that, contrast stretching is fed into the picture with the noise eliminated.

***Contrast Stretching:*** This technique increases the visibility of features by stretching the range of intensity values to a desired range of values to improve the contrast in $\left(N_{rem}\right)$ after the noise has been removed. The contrast stretched image is $\left(S_{cont}\right)$:

(13) $$S_{cont}=\left[S_{cont\left(1\right)},S_{cont\left(2\right)},S_{cont\left(3\right)},\ldots ,S_{cont\left(a\right)}\right]$$

where $S_{cont\left(a\right)}$ represents the $a^{th}$ contrast-stretched image. Then, these contrast-stretched images are sent to the convex hull operation.

***Convex Hull Lung Region Extraction:*** This is designed to focus on the ROI, removing irrelevant parts of the image. It is a set of pixels included in the smallest convex polygon that surrounds all white pixels in the input. In this study, the lung area is separated from contrast-stretched picture $\left(S_{cont}\right)$ by using the convex hull. Therefore, the separated lung region is expressed as $L_{reg}$, which goes to edge enhancement.

***Edge Enhancement:*** The boundaries of the lung structures are sharpened in this step, improving the visibility of important features by enhancing the edge contrast of $L_{reg}$. In this work, edge enhancement is performed by using the UMF. The fundamental idea behind the UMF is to improve the original image by scaling and highlighting a portion of it. The edges or high-passed pixels are removed throughout this filtering step:

(14) $$A_{ed}=\gamma_{rs}Q\left(L_{reg}\right)$$

where $A_{ed}$ is the augmented edge, $\gamma_{rs}$ represents the boundary mining kernel, and Q represents gray content. The improved picture, (E), is created using the UMF:

(15) $$E=Q+\eta A_{ed}$$

where η indicates the gain factor that determines the potency of $\left(A_{ed}\right)$.

### 3.2. Segmentation

The proposed approach ensures that the most relevant features can be extracted for further analysis by precisely segmenting the lung regions in the preprocessed images. After preprocessing, segmentation is conducted to produce enhanced image (E).In this work, the images are segmented into four parts by using our suggested algorithm named BRGS. It produces high segmentation accuracy. Using predetermined seed pixels, growth criteria, and stop conditions, BRGS divides the image by grouping pixels into a bigger region. However, it leads to shadows overlapping the vessels, tissue mass, and ribs in CT images. So, to overcome this problem, the knuckle point suggested in BRGS adopted by modified Bates distribution coati optimization algorithm (BD-COA). Here, the BD-COA is selected for its high metaheuristic property, which provides a conformity index by selecting the highest conformity value. However, the BD-COA incurs extensive computational complications. To address this issue, the BRGS is used to calculate a random number used in proposed algorithm. Therefore, the proposed model is named BRGS. Algorithm 1 presents the proposed transfer learning model named BRGS. In this algorithm, initially, enhanced image (E) is fed into the RGS process. Let the seed point selection be the starting stage of the RGS process, and in this work, seed point $\left(S_{E}\right)$, which is the knuckle point of the lung image, is selected by using the COA. The actions and habits of the coati (pronounced ko-AH-tee) served as the inspiration for the population-based metaheuristic algorithm known as COA. Let the coati for the algorithm be initialized as seed point $\left(S_{E}\right)$. The locality of coatis in the search space is first set at random:

(16) $$P_{x,y}\left(S_{E}\right)=lw_{y}+\varsigma •\left(up_{y}-lw_{y}\right)\;x=1,2,\ldots m\;,\;y=1,2,\ldots n$$

where $\left(P_{x,y}\left(S_{E}\right)\right)$ represents the position of the $x^{th}$ coati of the $y^{th}$ decision variable, with *lw* and *up* representing the upper and lower bounds of the $y^{th}$ decision variable. Here, the upper value for the coati is m, n denotes the number of decision variables, and ς denotes the random number calculated by using the BD. The BD represents the mean of random variables uniformly distributed from 0 to 1, which reduces the complication in the described model. Therefore, the random number is determined as:

(17) $$\varsigma =\frac{1}{\phi }\sum_{o=1}^{\phi }U_{0}$$

where $U_{0}$ represents unit interval ϕ. Then, the coati population is represented by

(18) $${\left[\begin{array}{c} P_{1}\left(S_{E}\right)\\ \vdots \\ P_{x}\left(S_{E}\right)\\ \vdots \\ P_{m}\left(S_{E}\right) \end{array}\right]}_{m\times n}={\left[\begin{array}{ccccc} P_{1,1}\left(S_{E}\right) & \cdots & P_{1,y}\left(S_{E}\right) & \cdots & P_{1,n}\left(S_{E}\right)\\ \vdots & \vdots & \vdots & \vdots & \vdots \\ P_{x,1}\left(S_{E}\right) & \cdots & P_{x,y}\left(S_{E}\right) & \cdots & P_{x,n}\left(S_{E}\right)\\ \vdots & \vdots & \vdots & \vdots & \vdots \\ P_{m,1}\left(S_{E}\right) & \cdots & P_{m,y}\left(S_{E}\right) & \cdots & P_{m,n}\left(S_{E}\right) \end{array}\right]}_{m\times n}$$

Because of where possible solutions are placed in the choice variable, different values for the problem’s fitness are assessed. The accuracy of categorization is the basis for evaluating the fitness value in this study. The benefit of fitness is provided by

(19) $$fit={\left[\begin{array}{c} fit\left(P_{1}\left(S_{E}\right)\right)\\ \vdots \\ fit\left(P_{x}\left(S_{E}\right)\right)\\ \vdots \\ fit\left(P_{m}\left(S_{E}\right)\right) \end{array}\right]}_{m\times 1}$$

The position of the coati is updated based on two behaviors, namely, the coati’s strategy when hunting for iguanas and the coati’s run-away stratagem against predators.

***Coati’s approach to taking on iguanas:*** Using a new position, the coati attempts to assault an iguana with this tactic. As a result, the coati’s posture when it emerges from the tree is described as

(20) $$P_{x,y}{\left(S_{E}\right)}^{1}=P_{x,y}\left(S_{E}\right)+\varsigma •\left(iguana_{y}-\vartheta •P_{x,y}\left(S_{E}\right)\right)\;\;\;\;\;\;\;\;x=1,2,\ldots \left(\frac{m}{2}\right),y=1,2,\ldots n$$

where ϑ represents the integer that is arbitrarily selected from [1,2]. Additionally, the coati’s movement on the ground in the search area is described as follows:

(21) $$P_{x,y}{\left(S_{E}\right)}^{1}=\left\{ \begin{array}{ll} P_{x,y}\left(S_{E}\right)+\varsigma •\left(iguana_{y}^{gnd}-\vartheta •P_{x,y}\left(S_{E}\right)\right) & ,\;fit_{iguana^{gnd}}<fit_{x}\\ P_{x,y}\left(S_{E}\right)+\varsigma •\left(P_{x,y}\left(S_{E}\right)-iguana_{y}^{gnd}\right) & ,\;else \end{array}\right.\;\;\;\;\;\;\;$$

In (21), $\;x=\left[\frac{m}{2}\right]+1,\left[\frac{m}{2}\right]+2,,\ldots m,y=1,2,\ldots n$, and $iguana_{y}^{gnd}$ represents the location of the iguana on the ground in the $y^{th}$ dimension, expressed as

(22) $$iguana_{y}^{gnd}=lw_{y}+\varsigma •\left(up_{y}-lw_{y}\right)$$

Therefore, the updated position is given by

(23) $$P_{x,y}\left(S_{E}\right)=\left\{ \begin{array}{ll} P_{x,y}{\left(S_{E}\right)}^{1} & ,\;fit_{x}^{1}<fit_{x}\\ P_{x,y}\left(S_{E}\right) & ,\;else \end{array}\right.$$

***Coati’s escape strategy from predators:*** In this strategy, an arbitrary location is generated near the position denoting the location of every coati:

(24) $$lw_{y}^{local}=\frac{lw_{y}}{tn},up_{y}^{local}=\frac{up_{y}}{tn},\;where\;tn=1,2,\ldots Tn$$

(25) $$P_{x,y}{\left(S_{E}\right)}^{2}=P_{x,y}\left(S_{E}\right)+\left(1-2\varsigma \right)•\left(lw_{y}^{local}+\varsigma \left(up_{y}^{local}-lw_{y}^{local}\right)\right)$$

where Tn represents the maximum number of iterations for (tn). Therefore, the updated position is

(26) $$P_{x,y}\left(S_{E}\right)=\left\{ \begin{array}{ll} P_{x,y}{\left(S_{E}\right)}^{2} & ,\;fit_{x}^{2}<fit_{x}\\ P_{x,y}\left(S_{E}\right) & ,\;else \end{array}\right.$$

Updating the population is performed repeatedly until the algorithm’s last iteration. Exactly like updating a position based on certain strategies, the seed point is selected using BD-COA. Therefore, the selected seed point is expressed as $\left(p_{seed}\right)$. Next, based on a gray-scale value for $\left(p_{seed}\right)$, the region-growing operator classifies the neighboring pixels. The distinction between these values will be used as a threshold to determine whether or not the pixel is a part of the ROI. If the distinction among the pixels $\left(p_{seed}\right)$ is fewer than the threshold, a pixel is labeled as part of the ROI; otherwise, the pixel is labeled as background. Finally, the four parts of segmented image $\left(S_{eg}\right)$ are given by

(27) $$S_{eg}=\left[S_{eg\left(1\right)},S_{eg\left(2\right)},S_{eg\left(3\right)},S_{eg\left(4\right)}\right]$$

**Algorithm 1:** BRGS**Input:** Enhanced image (E)**Output:** Segmented image $\left(S_{eg}\right)$

**Begin**
    **Initialize** enhanced image, maximum iterations Tn    **Select** seed point using COA     **For** all iterations **do**        **Initialize** position of coati using (16)         **Compute** random variable using BD         **Compute** population of coati using (18)         **Evaluate** fitness value         **For**
$x=1:\left(\frac{m}{2}\right)$            **Calculate** new position of coati using (20)             **Update** position using (21)         **End For**        **For** $x=\left[\frac{m}{2}\right]+1:m$            **Calculate** the position of the iguana using (22)             **Calculate** the new position of coati using (25)             **Update** the position of coati using (26)         **End For**    **End For**    **Return** seed point     **Compute** threshold     **Return** segmented image
**End**


The feature extraction procedure is completed after segmentation.

### 3.3. Feature Extraction

Feature extraction obtains gradient features and profile-based features, plus on-rib, on-vessel, and spectral flatness measures from segmented image $\left(S_{eg}\right)$. These features are explained below.

***Gradient features:*** The gradient characteristics from $\left(S_{eg}\right)$ are retrieved for each sub-image. The area under the modes is calculated based on the spacing between two modes, their relative statistical characteristics (such as their bimodality coefficient, skewness, and kurtosis), and the ratio of modes normalized by their separation. Therefore, the gradient features are expressed as $G_{Mea}$.

***Spectral flatness:*** It is based on the $\left(G_{Mea}\right)$ from each sub-image, spectral flatness measures the edginess of $\left(S_{eg}\right)$. It can be defined as the geometric mean divided by the arithmetic mean of the image’s Fourier coefficient magnitudes. Therefore, the spectral feature measure is expressed as $Sp_{flat}$.

***Attributes based on a profile:*** Profile-based characteristics are retrieved from the normalized smoothed magnitude of each sub-image of $\left(S_{eg}\right)$ and are described below.

- **Rib cross:** The profiles from the edge picture are taken to extract this feature. When a rib edge is present, the profile has a peak assigned score of prof(ϕ)=1.
- **Peak ratio:** This is obtained by calculating the maximum peak to minimum peak ratio and the average for each extracted profile.
- **Slope ratio:** This is obtained by calculating the profile’s first-order derivative and taking the average:
  (28) $$Slope_{\min\max}=\frac{\min\left(prof\left(diff\right)\right)}{\max\left(prof\left(diff\right)\right)}$$
- **Slope smoothness:** This characteristic determines how smooth the slope is. It is obtained by calculating the second-order derivatives for each profile, then the steepness value and the average. Therefore, the profile-based features are expressed as $prof_{fea}$.

***On-rib:*** This feature determines if there is a cancer it will assign it a feature value [50,56,57]. This entails rib edges being located. On the rib, cancer is suggested if the distance between the centroid and the segment is shorter than the inter-rib distance (the difference between the centroid and the rib). Based on the segment length, slope, and eccentricity parameters, on-rib characteristics are computed. Therefore, the on-rib features are expressed as $O_{rib}$.

***On-vessel:*** It is based on the length and eccentricity of these edges, the on-vessel characteristics are calculated. One should determine the lengths of all the vessel edges and then choose the two that have the longest lengths possible by calculating the length:

(29) $$Vessel_{1}=\frac{\left(\max leng_{1}*\max leng_{2}\right)}{\left(height*width\right)}$$

Then, one should determine the shortest distance between each edge and the sub-image’s center, choose the minimum distance (mindist) between them all, and obtain the inverse value:

(30) $$Vessel_{2}=\frac{1}{\min\left(\min dist\right)}$$

On-vessel features are expressed as $O_{vessel}$. Finally, the extracted features are given by

(31) $$Fea_{ext}=\left[G_{Mea},Sp_{flat},prof_{fea},O_{rib},O_{vessel}\right]$$

The feature selection procedure then uses these extracted characteristics as input [58,59].

### 3.4. Feature Selection

Feature extraction is conducted after preprocessing to extract the most pertinent data from the pictures. The variables that are extracted are important for diagnosing malignant lung nodules, and include texture, shape, intensity, and edge-based properties. These characteristics are chosen to make a substantial contribution to the proposed model’s performance, having been evaluated for their applicability to lung nodule detection and classification tasks.

After feature extraction, the most significant features are determined by using the proposed Algorithm 2 named BD-CST. CST is a numerical test that evaluates a feature event that is independent of the class value and quantifies the departure from the predicted distribution. It requires little processing time, and is limited to choosing the significance level randomly, which may affect the processing time. Hence, to overcome this problem, binomial distribution is proposed for choosing the significance level, which reduces the processing time. The steps in the BD-CST method are as follows:

(1) Hypothesis and your analysis plan.
(2) Analyze the sample data and forecast the outcome.

(1) **Specify the hypothesis and analysis plan:** The BD-CST model receives extracted characteristics, $\left(Fea_{ext}\right)$, as input. The hypothesis and then the analytical plan on how to use model data to support or refute the hypothesis are described. The following must be specified in the plan: significance rank and test technique.

In this study, binomial distribution was used to select the significance rank. The discrete probability distribution (binomial distribution) has two possible outcomes, and aids in shortening the model’s processing time. Consequently, the importance rank, (R), can be determined by

(32) $$R=\left(\begin{array}{l} e\\ \rho \end{array}\right)g^{\rho }h^{e-\rho }$$

where ρ is the number of successes; the number of trials is denoted by e, $g^{\rho }$ is the probability of success, and $h^{e-\rho }$ is the chance of failure. To determine if there is a significant association between two categorical qualities, the independent level is tested using the chi-square test.

(2) **Examine the sample data and predict the results**: The selected data must be examined at this stage to determine the test’s degree of freedom, the predictable frequency, the test value, and the probability value. One can figure out the degree of freedom, $\left(D_{fr}\right)$, as follows:

(33) $$D_{fr}=\left(\varpi -1\right)*\left(\zeta -1\right)$$

where ϖ denotes the level of one categorical variable, and ζ denotes the level of another categorical variable. The features, $\left(Fea_{sele}\right)$, are chosen as follows:

(34) $$Fea_{sele}=\frac{e*{\left(WZ-YX\right)}^{2}}{\left(W+Y\right)\left(X+Z\right)\left(W+X\right)\left(Y+Z\right)}$$

where W represents the number of times $Fea_{ext}$ and ζ co-occur, X delineates the number of times $Fea_{ext}$ appears without ζ, Y is how many times ζ appears without $Fea_{ext}$, and Z is the number of times neither ζ nor $Fea_{ext}$ occur.

So, we can say that our utilization is a combination of conventional image processing methods and deep learning architectures to extract characteristics from preprocessed lung CT scans for the PResNet lung carcinoma prediction, risk screening, and classification model.

**Surface analysis:** To describe the location and arrangement of pixel intensities within the lung tissue, features capturing texture patterns are extracted. Examples are GLCM features, local binary patterns (LBPs), and gray-level run-length matrix features. 

**Aspects of shape:** Geometric features are calculated to measure the morphological properties of any anomalies, like nodules or masses, in the lung region. These shape descriptors include circularity, eccentricity, and uniformity.

**Intensity histograms:** The distribution of pixel intensities inside the lung tissue is captured using histogram-based characteristics, which provide information about tissue density and homogeneity. These features include mean intensity, standard deviation, skewness, and kurtosis.

Using pre-trained Convolutional neural network (CNN) models, we extract important features from lung tomography scans using our proposed PResNet. These characteristics are essential for classifying and evaluating lung cancer risk. Here is a comprehensive explanation of the steps implemented and the features extracted.

*Edges and Contours:* The CNN’s first layers identify fundamental structural components, including edges, lines, and corners. These characteristics are essential for determining the limits of lesions and lung tissues. 

*Textures:* The early layers also capture patterns and textures in the lung images, such as areas that are smooth or rough. These are important for recognizing abnormal healthy tissue. 

*Shapes and Regions:* Nodules, masses, and other complex lung structures are detected by the CNN’s middle layers. This helps in the detection of abnormalities and abnormal growths.

*Intensity Variations:* At this stage, variations in pixel intensity that might indicate different tissue types or densities are tracked, which helps discriminate between different lung diseases.

The PResNet model’s accuracy and durability are significantly improved by the extracted features.

### 3.5. Classification

**Figure 2:**
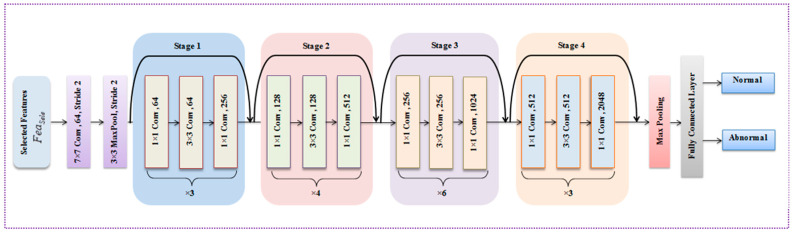
The architectural design of PResNet.

The PResNet classifier is a deep learning model that uses transfer learning to classify images as normal or abnormal. It also predicts the risk level (low or high) for each image that is categorized as abnormal [60]. After feature selection, the classification process determines whether selected features $\left(Fea_{sele}\right)$ are normal or abnormal. In this work, classification is performed by using a transfer learning-based PResNet algorithm [12], which significantly enhances the performance of the network with more layers. The classic ResNet uses skip connection, which connects the activation of the layer to further layers by skipping a layer in between that forms a residual block [61,62,63]. However, it is still affected by a low learning rate, and the kernel activation function leads to a high computation time. To overcome this, transfer learning (TL) is proposed. TL is basically a machine learning technique that employs a previously trained model as the basis for a model on a novel assignment. The system is strengthened and made more secure by employing the TL technique. The model’s effectiveness is increased by using the p-ReLU activation function. Hence, our model is named PResNet. Figure 2 demonstrates PResNet’s architecture.

The activation layer, convolution layer, pooling layer, and fully connected layer are the four layers that make up PResNet. Initially, $\left(Fea_{sele}\right)$ is specified as input for the convolution layer. During this process, feature mapping is performed to classify the output. The output of the convolution layer, $\left(con_{lyr}\right)$, is given by

(35) $$con_{lyr}=\mu \sum Fea_{sele}•w$$

The p-ReLU activation function generalizes the conventional rectifier linear unit and has a slope for negative values [59,64] where w indicates the weight; μ represents the p-ReLU activation function:

(36) $$\mu =\left\{ \begin{array}{ll} \mu & ,\;if\;\mu >0\\ J\mu & ,\;if\;\mu \leq 0 \end{array}\right.$$

where J represents the negative slope. After that, the outcome of the convolution layer is fed into the maximum pooling layer, $\left(pol_{lyr}\right)$, which reduces the dimension of the feature map by selecting relevant features:

(37) $$pol_{lyr}=\max\left(\frac{con_{lyr}-w}{\kappa }\right)$$

where *k* represents the stride length that decides the number of pixel shifts under the various weights. Then, the outcome of the maximum pooling layer used is specified for the fully connected layer that classifies the output of the given image. A fully linked layer’s output is determined as follows:

(38) $$full_{lyr}=\mu \left(pol_{lyr},w+pol_{lyr}\right)$$

**Algorithm 2:** BD-CST**Input:** selected features $\left(Fea_{sele}\right)$**Output:** Classified output as Normal or Abnormal**Begin**    **Initialize** selected features $\left(Fea_{sele}\right)$, weight w    **For** all training steps **do**        **Perform** convolution layer             $con_{lyr}=\mu \sum Fea_{sele}•w$        **Compute** p-ReLU function         **Perform** max pooling layer         **Process** fully connected layer             $full_{lyr}=\mu \left(pol_{lyr},w+pol_{lyr}\right)$    **End For**    **Return** classified output as Normal or Abnormal**End**

Finally, PResNet classifies the output as normal $\left(N_{mrl}\right)$ or abnormal $\left(AN_{mrl}\right)$. If the output is abnormal, a further risk screening process is carried out.

### 3.6. Risk Screening

Risk Classification: The model predicts whether the detected malignancy poses a low or high risk based on the features that were observed. This information helps with early diagnosis and suitable medical treatment.

Risk screening identifies the risk of harm and then minimizes the risk recognized. In this work, during the risk screening process [65,66,67] the on-rib features of abnormal images $\left(AN_{mrl}\right)$ were considered because the risk of carcinoma can easily be detected as high risk or low risk:

(39) $$Rk_{screen}=\left\{ \begin{array}{ll} Rk_{high} & ,\;if\;carcinoma\;\;present\;\\ Rk_{loe} & ,\;if\;carcinoma\;\;not\;present \end{array}\right.$$

where $Rk_{screen}$ represents the risk screening, $Rk_{high}$ denotes high risk, and $Rk_{low}$ denotes low risk. Finally, the patients were treated.

For the model to be successful, preprocessing methods must ensure that the original features in images are enhanced [29].

Visual Inspection: One should analyze a sample of images both before and after each preprocessing stage to make sure that features that are significant to the identification of lung cancer are preserved. It might be necessary to make changes to the preprocessing method if considerable loss is identified.

**Figure 3:**
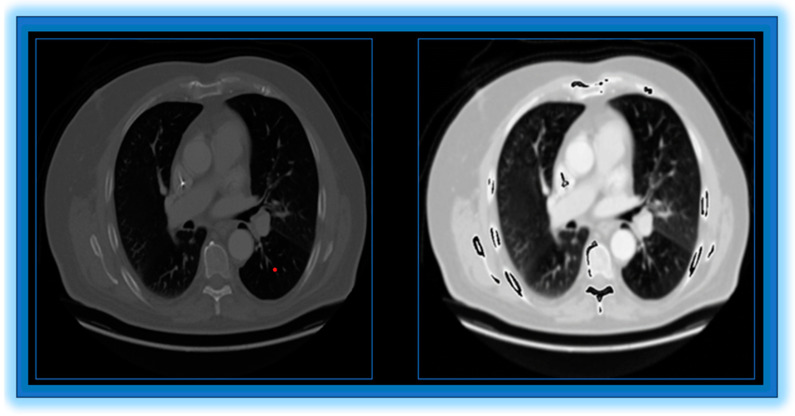
Left image, without preprocessing; right image, with preprocessing.

Figure 3 shows the result of preprocessing and without preprocessing.

Quantitative Evaluation: To evaluate the way preprocessing impacts image quality and feature preservation, quantitative measurements are used. Mean squared error (MSE), peak signal-to-noise ratio (PSNR), and the structural similarity index (SSIM) are examples of common metrics. These measures might offer quantifiable proof of an improvement in image quality.

The pre-existing models do not obtain the desired level of precision and speed for their particular application. The suggested model might be designed in a way that is more computationally efficient, requiring less memory, processing power, and energy consumption. Pre-existing models might not scale well with higher-resolution inputs and larger datasets, indicating the need for a new design with novel techniques (e.g., improved performance from new types of layers and activation functions). Pre-built models might lack the flexibility to adapt to changes in the dataset, while the proposed model might offer better adaptability.

## 4. Results and Discussion

This section evaluates the effectiveness of the suggested framework by contrasting its outcomes with those of other models already in use. The suggested paradigm is put into practice using the Python development environment. Data from the upper body, especially from the Chest CT Image Lung dataset, were gathered for the analysis.

### 4.1. Dataset Description

Using a carefully selected dataset of lung CT scans, we assessed the effectiveness of the PResNet model for lung cancer prediction and risk screening. A thorough description of the dataset is given in this section, together with information on its sources, the number of images, the planning stages, and prominent features. The Lung Image Database Consortium (LIDC) and the Image Database Resource Initiative (IDRI) are two publicly accessible medical image repositories from which the dataset for this study was retrieved. These databases offer a comprehensive collection of analyzed lung images for research, and are often utilized in medical imaging, with 3200 images from lung CT scans in the collections. Expert radiologists have contributed broad interpretations for every image in the dataset selected. Among the notations are lesion markings with ROIs for lung cancer indicated on lesions or nodules, and severity classifications for abnormal pictures (low risk and high risk) depending on how severe the malignancy was. Data augmentation methods were used to enhance the model’s robustness. Rotation imitates points of view, with images rotated at different angles. Scaling improved the model’s ability to generalize, and images were scaled to produce size changes. Flipping horizontally and vertically improved the dataset’s diversity. Translation imitated different positions, with images translated or shifted in different directions. The dataset was split into testing and training sets in order to assess how well the suggested model performed. PResNet was trained using 2560 images (80% of the selected dataset) and tested with 640 images (20% of the dataset) to validate the usability of the model. The following standard metrics were used to assess the model’s performance. Accuracy is the proportion of correctly classified images out of the total number of images. Precision is the percentage of actual positive outcomes among every positive result that the model predicted. Recall is the percentage of positive results in the dataset that are true positives.

The data include one folder for normal cells and three distinct types of lung cancer: adenocarcinoma, large cell carcinoma, and squamous cell carcinoma. The main folder containing all of the files is called *data*, and inside it are folders called *test*, *train*, and *valid*. 

Features such as texture patterns, edge details, nodule forms, and density changes were retrieved by pre-trained CNN models. These characteristics are essential for determining the severity of the cancer and differentiating between normal and malignant lung tissues. To improve the resilience of the model, data augmentation techniques such as rotation, scaling, flipping, and translation were used.

**Figure 4:**
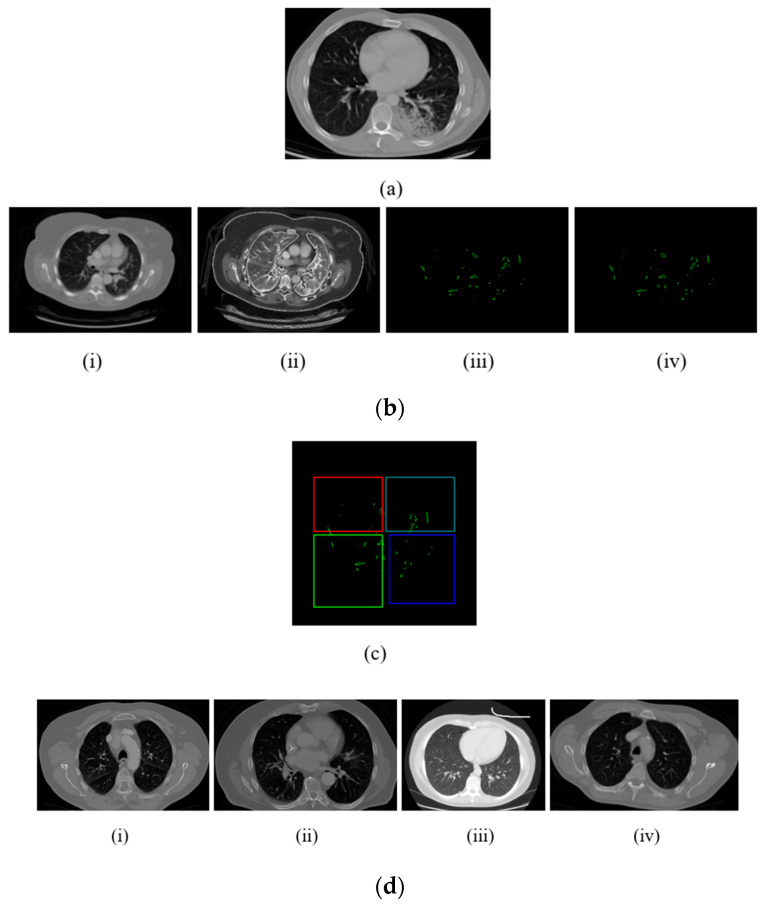
Sample images of the planned model: (**a**) input picture, (**b**) preprocessed images, (**c**) segmented images, and (**d**) classified output.

Figure 4 presents sample images from the proposed methodology. The input image is shown in (a). The input image preprocessed by using the I-ADF and the UMF is shown in (b),where part (i) is the image with noise removed, part (ii) is the contrast-stretched image, part (iii) is the convex hull image, and part (iv) shows the edge enhancement. The segmented image is (c), and the classified output is d(i) adenocarcinoma, d(ii)large cell cancer, and d(iii) squamous cell cancer, with d(iv) showing a normal scan (no carcinoma).

With the 80:20 split to prove the robustness and dependability of our evaluation, a thorough evaluation is guaranteed, overfitting is reduced, and the model’s performance in various situations is accurately measured.

### 4.2. Classification Analysis

The performance assessment of PResNet confirmed the following parameters: error rate, training time, recall, sensitivity, specificity, f-score, false negative rate (FNR), false positive rate (FPR), false rejection rate (FRR), and accuracy. The results were then contrasted with those from other current models, including ResNet, a CNN, a DNN [46,60], and an artificial neural network (ANN) [35,66].

Table 1 lists the performance of the proposed model compared with the existing models in terms of f-score, sensitivity, specificity, recall, accuracy, and precision. Higher values indicate better performance. The accuracy of the suggested model was 98.21%, which is higher than the existing models: 94.66% (ResNet), 93.01% (the CNN), 92.38% (the DNN), and 87.26% (the ANN). Additionally, the suggested framework surpassed the current framework for accuracy, recall, sensitivity, specificity, and f-score. Consequently, we concluded that the proposed model outperformed earlier models.

**Figure 5:**
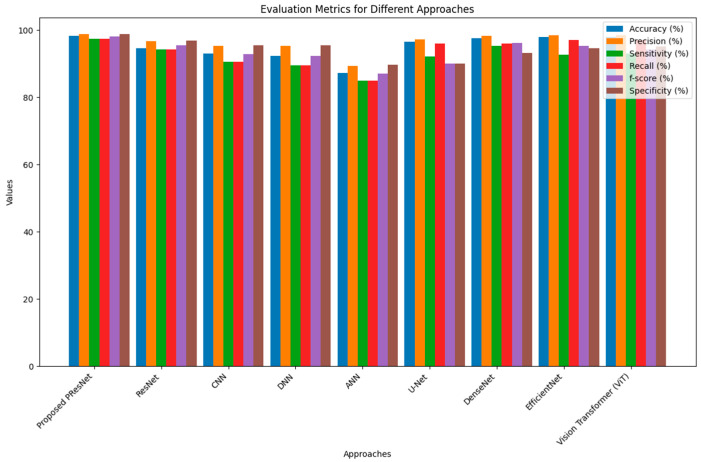
Evaluation of the proposed model and state-of-the-art models.

Figure 5 is a comparative evaluation of the proposed model and already-built models in terms of the FNR, FPR, FRR, and error rate. A lower FNR, FPR, FRR, and error rate indicates better performance. The suggested model’s FNR was 0.02531, compared to FNR values attained by the existing models of 0.05844 (ResNet), 0.09493 (the CNN), 0.1055 (the DNN), and 0.15094 (the ANN). Similarly, the FPR, FRR, and error rates attained by the proposed model were 0.0128, 0.0127, and 0.0191, which demonstrate its improved performance.

**Figure 6:**
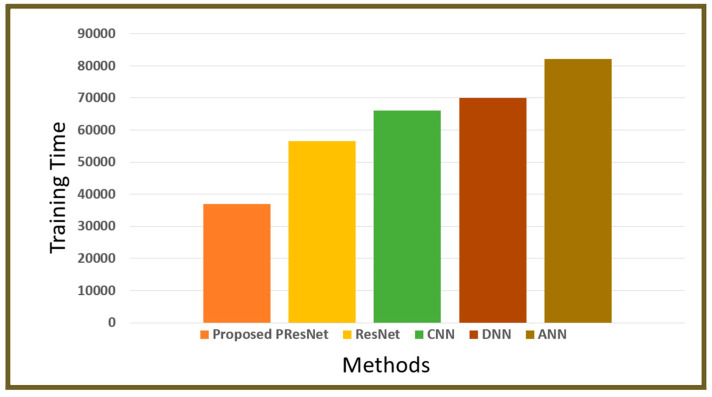
Training time of the proposed model versus state-of-the-art models.

Figure 6 displays the training times for the proposed model and current models. Here, the proposed model’s training time was 36,938.55 ms, while the training timesfor the other models were 56,639.28 ms (ResNet), 66,016.98 ms (the CNN), 70,078.52 ms (the DNN), and 82,063.17 ms (the ANN), suggesting that our model is more effective at classifying data.

### 4.3. Performance Analysis of Segmentation

Dice score measurements were used to validate the performance of the B-RGS. Then, the outcome was compared with pre-existing models like RGS, Wise Sliding (WS) window, the Ostu (OS) algorithm, and the K-Means Algorithm (KMA).

**Figure 7:**
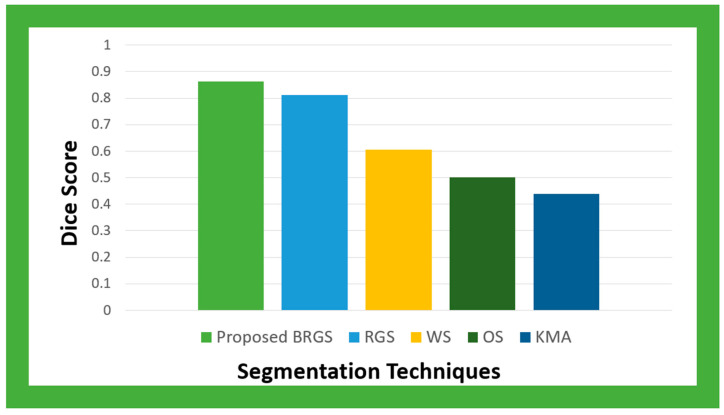
The Dice score values of the proposed model and pre-existing models.

Figure 7 displays the Dice score values for the proposed model and the pre-existing models. When evaluating the effectiveness of picture segmentation techniques, the Dice score is utilized. The models achieved Dice score values of 0.81291 (RGS), 0.60652 (the WS window), 0.50240 (OS), and 0.4385 (the KMA), compared to the proposed model’s 0.86173. The findings show that the recommended approach performs better at data segmentation.

### 4.4. Analysis of Noise Removal

The I-ADF’s performance was validated in terms of the MSE, PSNR, and SSIM and then contrasted with other techniques, including ADF, the Gilbert filter (GF), a median filter (MF), and a bilateral filter (BF).

The performance of the planned model versus those of the pre-existing models for MSE, PSNR, and SSIM are shown in Table 2.

**Figure 8:**
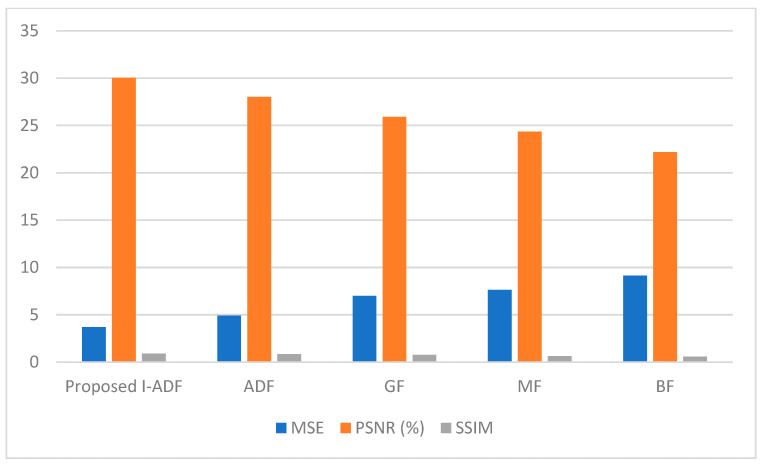
Comparison of noise filters.

Figure 8 shows a lower MSE and higher PSNR and SSIM due to the better performance of the proposed model. The MSE value attained by the proposed model was 3.710, which was lower than that of the pre-existing models. Similarly, the PSNR and SSIM values attained by the proposed model were 30.033% and 0.8933, respectively; they demonstrate that the model performed better. The I-ADF is therefore more effective at removing noise.

Table 3 contrasts the PResNet accuracy with models from the literature survey.

**Figure 9:**
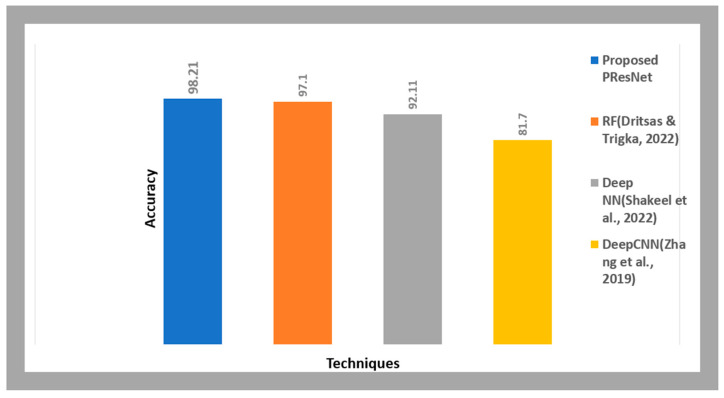
Comparative analysis for accuracy of our model and literature survey models.

PResNet’s accuracy was greater than that of the literature survey models, which achieved 97.1% (RF), 92.11% (the deep NN), and 81.7% (the deep CNN), as shown in Figure 9. As a result, we conclude that the proposed model predicts lung cancer more accurately.

For lung cancer prediction and risk screening, the ANN, the DNN, the CNN, and ResNet were selected based on performance, computational efficiency, robustness, and simplicity, even though they are more sophisticated. Our model offers the best possible combination of precision, effectiveness, and usability, which makes it optimal for medical purposes involving the early identification of lung cancer.

Comparing the suggested PResNet model with previous and advanced ones, significant improvements in accuracy, precision, and recall were observed. Significant results include a 20% reduction in model parameters, a 28% dropin inference time, and an accuracy improvement of 3.3%. The actual usefulness of the model is demonstrated by real-world applications, particularly medical imaging.

## 5. Conclusions

In this research, we suggested an effective process for the early prediction of lung carcinoma. Our proposed system categorizes lung CT pictures as normal or abnormal. The experimental results prove that the proposed model is more efficient than state-of-the-art models, achieving the best accuracy, precision, and recall of 98.21%, 98.71%, and 97.465, respectively. In addition, the suggested model performed well. The results prove it is more accurate at predicting lung carcinoma in a timelier manner. In the future, our work may predict the types of lung cancer and their severity by using advanced techniques with more efficiency.

## Figures and Tables

**Table 1:** Comparative analysis of the proposed model and other current models.

Approaches	Accuracy (%)	Precision (%)	Sensitivity (%)	Recall (%)	f-Score (%)	Specificity (%)
**Proposed PResNet**	98.21	98.71	97.46	97.46	98.08	98.71
**ResNet**	94.66	96.66	94.15	94.15	95.39	96.87
**CNN**	93.01	95.33	90.50	90.50	92.85	95.54
**DNN**	92.38	95.36	89.44	89.44	92.30	95.45
**ANN**	87.26	89.40	84.90	84.90	87.09	89.67
**U-Net**	96.50	97.20	92.10	96.00	90.02	90.00
**DenseNet**	97.60	98.30	95.30	96.00	96.21	93.22
**EfficientNet**	97.85	98.45	92.60	97.10	95.22	94.65
**Vision Transformer (ViT)**	97.95	98.50	93.00	97.00	94.23	95.12

**Table 2:** The proposed and the pre-existing models’ MSE, PSNR, and SSIM.

Method	MSE	PSNR (%)	SSIM
**Proposed Model**	3.7105	30.0334	0.8933
**ADF**	4.9105	28.0334	0.8533
**GF**	7.0002	25.9047	0.7703
**MF**	7.6316	24.3380	0.6440
**BF**	9.1208	22.1738	0.5784

**Table 3:** Accuracy comparison of PResNet versus models from the literature.

Techniques	Accuracy
Proposed PResNet	98.21
RF	97.1
Deep NN	92.11
Deep CNN	81.7

## Data Availability

Not applicable.
